# Recognition of single upper limb motor imagery tasks from EEG using multi-branch fusion convolutional neural network

**DOI:** 10.3389/fnins.2023.1129049

**Published:** 2023-02-22

**Authors:** Rui Zhang, Yadi Chen, Zongxin Xu, Lipeng Zhang, Yuxia Hu, Mingming Chen

**Affiliations:** Henan Key Laboratory of Brain Science and Brain-Computer Interface Technology, School of Electrical and Information Engineering, Zhengzhou University, Zhengzhou, China

**Keywords:** single upper limb motor imagery, deep learning, brain-computer interface (BCI), convolutional neural network (CNN), feature fusion

## Abstract

Motor imagery-based brain-computer interfaces (MI-BCI) have important application values in the field of neurorehabilitation and robot control. At present, MI-BCI mostly use bilateral upper limb motor tasks, but there are relatively few studies on single upper limb MI tasks. In this work, we conducted studies on the recognition of motor imagery EEG signals of the right upper limb and proposed a multi-branch fusion convolutional neural network (MF-CNN) for learning the features of the raw EEG signals as well as the two-dimensional time-frequency maps at the same time. The dataset used in this study contained three types of motor imagery tasks: extending the arm, rotating the wrist, and grasping the object, 25 subjects were included. In the binary classification experiment between the grasping object and the arm-extending tasks, MF-CNN achieved an average classification accuracy of 78.52% and kappa value of 0.57. When all three tasks were used for classification, the accuracy and kappa value were 57.06% and 0.36, respectively. The comparison results showed that the classification performance of MF-CNN is higher than that of single CNN branch algorithms in both binary-class and three-class classification. In conclusion, MF-CNN makes full use of the time-domain and frequency-domain features of EEG, can improve the decoding accuracy of single limb motor imagery tasks, and it contributes to the application of MI-BCI in motor function rehabilitation training after stroke.

## 1. Introduction

The brain-computer interface (BCI) establishes a channel for information exchange between the human brain and the outside world. It decodes the user’s intent through reading and analyzing brain signals (Wolpaw et al., 2002), and has been linked to a wide range of devices, including the use of spellers, wheelchairs, robotic arms and robotic exoskeletons (Kaufmann and Kubler, 2014; Kwak et al., 2015; He et al., 2018; Kim et al., 2018; Penaloza and Nishio, 2018; Yu et al., 2018; Jeong et al., 2019; Yao et al., 2022). Among the various types of BCI paradigms, MI-BCI is one of the most important one because it has potential clinical application value. MI is a mental process that mimics motor intention without actually eliciting motor behavior. It is an actively evoked EEG signal that has high application values in the field of neurorehabilitation because it can independently elicit potential activity in motor-related brain regions without external stimulation (Pfurtscheller and Neuper, 2001). The MI-BCI detects the user’s motor intentions by capturing the potential changes, and the output command could be used to control functional electrical stimulation (FES), exoskeletons, or other rehabilitation assistive equipment (Biasiucci et al., 2018; Zhao et al., 2022). Thus MI-BCI is valuable in the medical rehabilitation pathway for patients with motor dysfunction through the provision of active rehabilitation training (Jeong et al., 2019; Romero-Laiseca et al., 2020). A large number of studies have shown that the addition of MI-BCI helps to promote the recovery of motor function and improve the quality of life of patients (Cervera et al., 2018; Yuan et al., 2021).

The majority of current researches on motor imagery EEG signal recognition focuses on movements of different body parts, such as the tongue, hands, and feet. These studies have produced excellent results, but it is uncommon to find studies on motor imagery EEG signal recognition of tasks that involve the same side of the limb. It is well known that limb motor dysfunction caused by stroke is often unilateral. In BCI-based rehabilitation training, motor imagery tasks using unilateral limbs are more natural and intuitive than motor imagery tasks between different body parts (Tavakolan et al., 2017; Ubeda et al., 2017). However, the classification of single limb motor imagery is more difficult and complex than that of different parts of the body, because similar brain regions are activated when performing different motor tasks for unilateral limbs (Bigdely-Shamlo et al., 2015; Jas et al., 2016; Taulu and Larson, 2021). Considering the low spatial resolution of EEG, it is not feasible to use the algorithms for multi-limb motor imagery EEG recognition to identify unilateral limb motor imagery EEG.

The issue of unilateral limb movement task recognition has begun to be focused on by some researchers in recent years. Edelman et al. (2016) reported that source space analysis can improve the classification accuracy of wrist movements, four different movements of the right hand (i.e., flexion and extension of the arm; left and right rotation of the wrist) were recognized with a classification accuracy of 81.4%. Ofner et al. (2017) encoded motor imagery tasks for the right hand into the time domain of low-frequency EEG signals to classify six different movements, including elbow flexion/extension, forearm left/right rotation, and hand opening/closing, and achieved an accuracy of 27%. A novel classification strategy using the combination of EMG and EEG signals was proposed by Li et al. (2017). They recognized a variety of upper limb movements such as hand open/close and wrist pronation/supination, and results showed that the classification performance achieved by the fusion features of EMG and EEG signals is significantly higher than that obtained by a single signal source of either EMG or EEG across all subjects. Loopez-Larraz et al. (2018) further used EMG activity as a complementary information to EEG to detect the motor intention, and also found that the fusion features achieved higher classification accuracy than EEG or EMG-based methods.

The end-to-end deep learning techniques provide a new development path for the recognition of motor imagery EEG. Inspired by the filter bank common spatial pattern (FBCSP), Schirrmeister et al. (2017) proposed three types of CNN-based models for motor imagery classification based on the number of layers. Jeong et al. (2020b) proposed a hierarchical flow convolutional neural network model consisting of a two-stage CNN for extracting relevant features for multi-class tasks and decoding arm rotation tasks. Zhang X. et al. (2019) proposed a network model CNN-LSTM, the motor imagery EEG data were spatially filtered by the FBCSP algorithm to extract the spatial domain feature information from the original data at first, then the extracted feature were fed into the CNN, and the final classification was performed by the LSTM. Cho et al. (2021) proposed a two-stage network structure called NeuroGrasp, which used six different CNN-BLSTM networks to implicitly map EEG signals to six muscle synergy features based on EMG and generated kinematic images corresponding to the EMG signals based on the extracted features. In the second stage, the generated images and real EMG features are used together as SiamNet network input to train the model, so as to realize the classification of single upper limb motor imagery tasks.

Most of the motor imagery EEG decoding methods based on deep learning used a single type of feature, including raw EEG signals, time-frequency maps, and power spectral density features. However, a single feature input often cannot fully and effectively mine the information related to motor imagery in EEG. Inspired by multimodal classification models, we proposed a multi-branch fusion convolutional network model (MF-CNN) for solving the classification problem of a single upper limb movement imagery task, which takes the EEG signals and the corresponding time-frequency maps as inputs simultaneously to make full use of the time-domain, frequency-domain and time-frequency-domain features of the EEG signal. The original EEG signal has high-resolution temporal information, and the discriminative features can be extracted by spatio-temporal convolution, while the two-dimensional time-frequency map contains rich time-frequency domain and spatial information. In this work, we first extracted the features of the above two inputs independently using two CNNs and then performed fusion classification, and the test results on the single upper limb motor imagery dataset showed that the proposed model achieved higher classification accuracy than single-input CNN.

## 2. Materials and methods

### 2.1. Datasets

The EEG data used in this work is the “Multimodal signal dataset for 11 intuitive movement tasks from single upper extremity during multiple recording sessions” from the Giga DB dataset completed by Jeong et al. (2020a). The dataset included intuitive upper limb movement data from 25 subjects, who were required to perform three types of motor tasks in a total of 11 categories, including 6 directions of arm extension movement (up, down, left, right, front, back), 3 kinds of object grasping action (cup, card, ball) and 2 kinds of wrist-twisting action (left rotation, right rotation), each type of movement was randomly executed 50 times, corresponding to 11 movements designed to be associated with each segmental movement of the arm, hand, and wrist, rather than continuous limb movements. The dataset included not only EEG data but also magnetoencephalography (EMG) and electrooculogram (EOG) data, which are collected simultaneously in the same experimental setting while ensuring no interference between them. The data were acquired using a 60-channel EEG, 7-channel EMG, and 4-channel EOG. In the current work, only motor imagery EEG data were used, the EEG sensors were placed according to the international 10–20 system, and the sampling rate was set as 2,500 Hz. Our goal is to classify the motor imagery EEG of the three types of actions, so we selected forward extension of the arm, grasping the cup, and rotation of the wrist to the left from the three types of actions for the following study.

### 2.2. Algorithm framework

**FIGURE 1:**
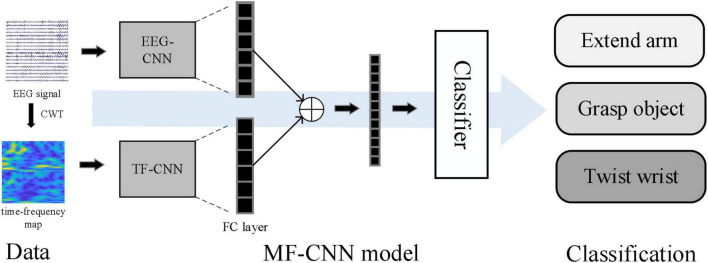
Workflow of the proposed algorithm.

The workflow of the algorithm was shown in Figure 1. The time-frequency maps were firstly obtained by continuous wavelet transform (CWT) method, then both the EEG signals and the corresponding time-frequency maps were sent to the MF-CNN model, which consisted of two CNN network branches. After the process of convolution and pooling, the output features from the two branches were fused and combined into a one-dimensional vector. Finally, the one-dimensional feature vector was sent into a classifier to obtain the prediction results.

### 2.3. EEG signal pre-processing

When subjects were preparing or performing motor tasks, event-related desynchronization (ERD) and event-related synchronization (ERS) can be observed in the sensorimotor cortex of the brain (Pfurtscheller and da Silva, 1999; McFarland et al., 2000). Therefore, we selected 20 EEG channels on the sensorimotor cortex region to analyze (including FC1-6, C1-6, CP1-6, CZ, and CPZ). The selected EEG data were band-pass filtered within 8–30 Hz (Sreeja et al., 2017) and downsampled to 250 Hz. All the 4 s of EEG data during the motor task of a single trial were intercepted for subsequent processing, thus the EEG segment of each trial could be defined as a 20 × 1,000 matrix, where 20 was the number of channels and 1,000 was the length of the data. The preprocessed EEG signals were used as input for the EEG-CNN branch and the time-frequency map conversion.

**FIGURE 2:**
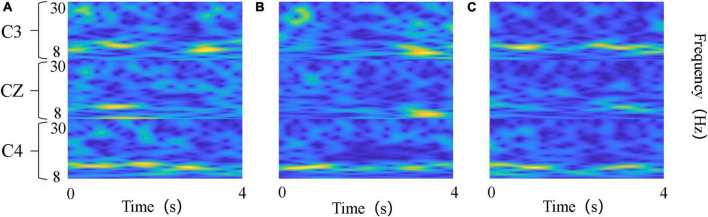
Time-frequency maps of the three kinds of tasks. **(A)** Left wrist rotation; **(B)** cup grasping; **(C)** forward arm extension. The abscissa denotes time points, and the ordinate denotes frequency bands.

In terms of time-frequency map transformation, Tabar and Halici (2017) proposed a method based on the short-time Fourier transform (STFT) to extract time-frequency features and constructed a three-channel stacked time-frequency map for subsequent classification. However, the time window of the STFT algorithm is fixed, so the time-frequency resolution is also fixed, which causes the problem of incompatibility between the time resolution and the spectral resolution. To solve this problem, wavelet transform based time-frequency analysis methods have been widely introduced to EEG signal feature extraction (Zhang et al., 2021). The wavelet transform replaced the infinite-length triangular function with a finite-length wavelet basis with attenuation, which made the window width inconsistent and thus enabled better local feature extraction. We chose Morlet wavelet as the basis function for the wavelet transform. As a single-frequency complex sinusoidal function under Gaussian envelope Morlet wavelet is the most commonly used complex-valued wavelet. Because it has a better local resolution in the time and frequency domain, it is often used in the decomposition of complex signals and time-frequency analysis (Lee and Choi, 2019). The features extracted from EEG signals through CWT include time and frequency information and are finally converted into two-dimensional time-frequency maps. Figure 2 showed the example time-frequency maps of the three channels C3, C4, and CZ.

Since the conversion of the time-frequency map is generated for each channel individually, the 20 EEG channels we used could not all be combined into one image. And if only a few channels were selected, a lot of helpful information would be lost. To effectively utilize the information of each channel, we preprocessed the data to extract features and used CSP to filter the 20 channels of EEG signals in the spatial domain to obtain “virtual channels,” and then generated the time-frequency maps. The basic principle of CSP is to find a set of optimal spatial filters for projection by diagonalizing matrices so that the difference in variance values between the two types of signals is maximized (Ramoser et al., 2000). For the three classification tasks we used the “One vs. Rest” strategy to extend the CSP to achieve multi-class CSP feature extraction (Dornhege et al., 2004). The spatially filtered EEG can be calculated as:


(1) $$Z_{M\times N}=W_{M\times M}\,E_{M\times N}$$

where *W* is the projection matrix of CSP, *M* is the number of EEG data channels; *N* is the data length; *E* is the EEG data matrix; *Z* is the obtained EEG on “virtual channels.”

**FIGURE 3:**
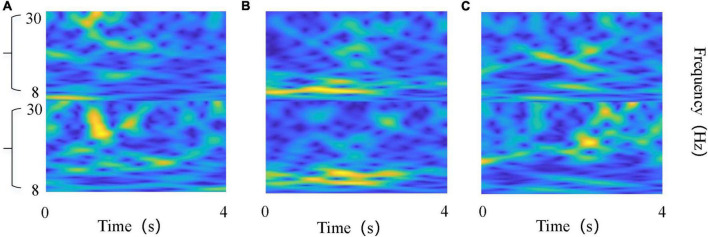
Time-frequency maps of the EEG on “virtual channel”. **(A)** Left wrist rotation; **(B)** cup grasping; **(C)** forward arm extension. The abscissa denotes time points, and the ordinate denotes frequency bands.

The information of the feature matrix generated by the CSP algorithm is not equivalent, and the feature information is mainly concentrated in the head and tail of the feature matrix, while the middle feature information is not obvious and can be ignored. Therefore, the first m rows and the last m rows (2 m < M) of *Z*_*M × N*_ were usually selected. In this work, we chose *m* = 1, that is, the first and the last row of *Z*_*M × N*_ were selected to calculate the time-frequency map. The CWT was applied to the spatially filtered EEG data during the 4 s motor imagery to obtain time-frequency maps, and the maps were then saved as images with a resolution of 64 × 64. Such procedures were applied to all trials, and finally the motor imagery time-frequency map dataset was obtained. An example was shown in Figure 3.

### 2.4. Structure of MF-CNN

The classification of motor imagery EEG signals using deep learning networks based on CNN has proven successful and has good feature extraction capabilities (Lee and Kwon, 2016; Zhang P. et al., 2019). The common CNN models include convolutional layer, pooling layer, activation function, and fully connected layer. The convolution in the network is a local operation that can extract the deep features of the input signal through the kernel function, then the feature information can be obtained by the operation of each layer of the CNN model. In the convolution phase, the network input is convolved with the convolution kernel, and then the activation function *f(a)* is used to output the feature maps, which can be expressed for each convolution layer as:


(2) $$h_{i\,j}^{k}=f\,\left(a\right)=f\,\left({\left(W^{k}*x\right)}_{i\,j}+b_{k}\right)$$

where *x* represents the input data, *W*_*k*_is the weight matrix of the *k*_*th*_ convolution kernel, *b_k_* corresponding to the deviation of the convolution kernel *k*, *i* and *j* denote the number of adjacent convolutional layers.

In the current work, the ReLU function was chosen as the activation function (Clevert et al., 2015), and it was defined as follows:


(3) $$f\,\left(a\right)=R\,e\,L\,U\,\left(a\right)=l\,n\,\left(1+e^{a}\right)$$

The main purpose of the fully connected layer in a CNN is classification. To merge the features acquired from the previous side, each node in the fully connected layer is connected to full nodes in the preceding layer. After a number of prior convolutions, it can combine the local information with category differentiation, and the output of the final fully-connected layer is then sent to the classifier to output the prediction result.

**FIGURE 4:**
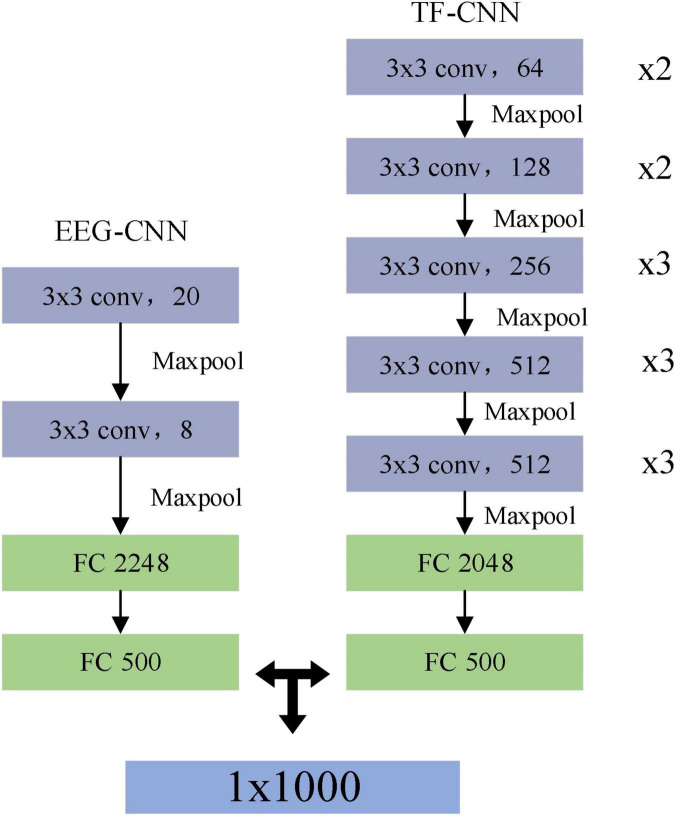
Structure of multi-branch fusion convolutional neural network (MF-CNN) model.

Figure 4 showed the network structures of MF-CNN proposed in this study, it extracted the features of the raw EEG data and the time-frequency map simultaneously by using two CNN branches, and could obtain more comprehensive information hidden in the motor imagery EEG.

The EEG-CNN branch extracted spatial and temporal features from the raw EEG data, the dimensionality of the input EEG signal was 20 × 1,000 (channels × points). The input was successively fed through a feature extraction module made up of two convolutional layers and a maximum pooling layer in this branch. A one-dimensional convolutional kernel along the horizontal axis was used to extract the features of each channel to obtain the feature map as the output of this layer. The size of the convolution kernel was set to 3 × 1, and the step size was 1. After convolution, a feature map of the form *N*_*w*_ × *N*_*f*_ could be obtained, where *N_w_* is the vector and *N_f_* is the number of convolution kernels. Then, the data from the convolutional layer was downsampled using the pooling layer, which set a kernel size of 2 × 1 and a step size of 2. Subsequently, the fully connected layer flattens the features extracted through the convolutional layer.

The TF-CNN branch performed feature extraction on the input time-frequency map, and the size of the time-frequency map was 64 × 64 × 3, which represented an RGB image of size 64 × 64. VGG16 was used as the basic network framework in this branch (Zhao-Hong et al., 2019), the main feature of which was the inclusion of convolutional kernel computation and feedforward structure. It contained 16 hidden layers (13 convolutional layers and 3 fully connected layers), the convolutional part used a convolutional kernel of size 3 × 3 with a step size of 1, and a max pooling layer of size 2 × 2 with a step size of 2.

In the model training phase of the above two branches, the parameters of each network layer were updated using the Adam optimizer with β_1_ 0.9 and β_2_ 0.999, with an initial learning rate of 0.01.

### 2.5. Feature fusion method

Generally, fusion methods can be applied in two different ways: decision-level fusion and feature-level fusion. Decision-level fusion first trains different modalities with different models and then fuses the results of multiple model outputs. Feature-level fusion combines two or more feature vectors to construct a single feature vector to include more information (Zhang P. et al., 2019; Hatipoglu Yilmaz and Kose, 2021). In this study, feature-level fusion was selected. Before the feature fusion, the individual feature vector must have sufficient relevant features in order to provide a good classification model and achieve high classification performance. In CNN, the fully connected layer can integrate local information into global features for classification, which contains enough information. In addition, the output dimension of the last fully connected layer is consistent with the category of the sample, and the obtained information has been compressed, so it is not appropriate to serve as the final feature vector. Therefore, we chose to use the penultimate fully connected layer of these two branch networks as the fusion layer, and fused their outputs as the extracted features. Suppose the output feature vector of the EEG-CNN branch was *A* = {*a*_1_,⋯,*a*_*m*_}, where *m* is the length of *A*, the feature vector output from the TF-CNN branch was *B* = {b_1_,⋯,b_n_}, where *n* is the length of *B*. Then the fusion feature vector could be defined as *C* = {a_1_,⋯,a_m_, b_1_,⋯,b_n_}, and it is fed into the support vector machine (SVM) to complete the classification finally.

### 2.6. Performance evaluations

The classification accuracy was used as an evaluation criterion to compare the model’s performance, which was calculated as follows.


(4) $$A\,c\,c\,u\,r\,a\,c\,y=\frac{T_{P}+T_{N}}{T_{P}+T_{N}+F_{P}+F_{N}}$$

where *T_P_* was the true-positives field in the confusion matrix, *T_N_*was the true-negatives field, *F_P_* was the false-positives field in the confusion matrix, *F_N_*was the false-negatives field. It indicates the probability of correct prediction in all samples. In this paper, we compared the accuracy of six algorithms, including our proposed MF-CNN, the two single-branch CNNs (EEG-CNN and TF-CNN), EEGNET (Lawhern et al., 2018), ALEXNET (Iandola et al., 2016), and the classical CSP. EEG-CNN, EEGNET, and CSP used EEG signals as inputs, which are pre-processed in the same procedures as described in (section “2.3. EEG Signal pre-processing”). TF-CNN and ALEXNET used time-frequency maps as input for image classification.

In addition, we calculated kappa values (Tabar and Halici, 2017).


(5) $$k\,a\,p\,p\,a=\frac{P_{0}-P_{e}}{1-P_{e}}$$

where *p_0_* represents the average classification accuracy and *p_e_*represents the random classification accuracy for the n-class classification task.

## 3. Results

In this work, three-class and binary-class classification (grasp object vs. extend arm) test tasks were carried out separately to verify the performance of the proposed algorithm. The classification accuracies were calculated by using the five-fold cross-validation strategy, each subject’s EEG data was divided into five equal subsets, one of which was randomly chosen as the testing dataset and the other subsets served as the training dataset. Such procedures were repeated five times, and the average accuracy was determined as the final classification accuracy. The three sessions for each subject were tested separately.

In order to verify the advantages brought by the dual-branch CNN, we compared the classification performance of MF-CNN model and single-branch CNN model. The single-branch CNN model was set up as an EEG-CNN branch for processing raw EEG signals and a TF-CNN branch for processing the time-frequency maps. The network architectures of these two single-branch CNN models were same as the EEG-CNN and TF-CNN branches in MF-CNN.

**FIGURE 5:**
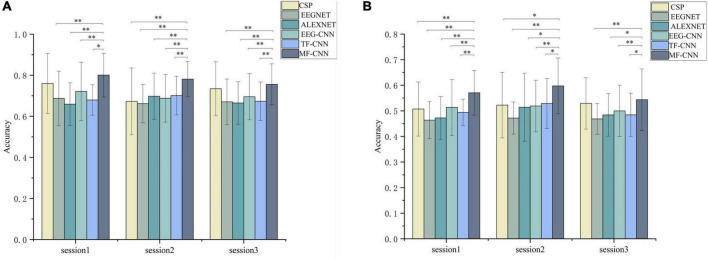
Comparison of the average classification results on the three sessions. **(A)** Binary-class classification experiment, **(B)** three-class classification experiments. *Denotes *p* < 0.01 and ^**^denotes *p* < 0.001 (paired *t*-test).

Figure 5 showed the classification results of the 25 subjects, the average classification accuracies of the single EEG-CNN branch were 70.8 and 51.08% separately for the binary-class and three-class classification experiments, while the single TF-CNN branch achieved 68.4 and 50.24%, respectively. It indicated that discriminative feature information can be extracted by the two kinds of single CNN branches. The accuracy obtained was higher than EEGNET and ALEXNET, but lower than CSP. After merging the features obtained from the two branches, MF-CNN achieved average accuracies of 78.52 and 57.06% for the two classification experiments, both of which were higher than that of the single CNN branch model, and also higher than CSP, EEGNET and ALEXNET.

The statistical analysis was further performed between the four algorithms using paired *t*-test. The results demonstrated that the accuracies achieved by MF-CNN were significantly higher than that of EEG-CNN and TF-CNN in all sessions. In addition, the accuracy of MF-CNN is higher than that of the deep learning algorithms EEGNET and ALEXNET used as comparisons.

**FIGURE 6:**
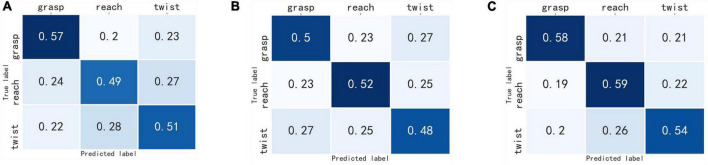
Confusion matrix of the three-class classification experiment. **(A)** EEG-CNN, **(B)** TF-CNN, **(C)** MF-CNN.

**FIGURE 7:**
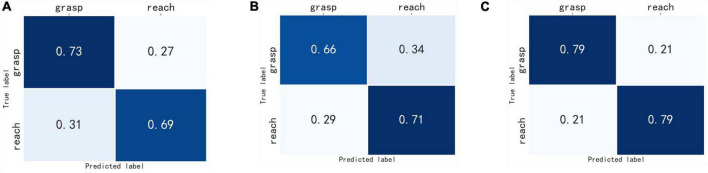
Confusion matrix of the binary-class classification experiment. **(A)** EEG-CNN, **(B)** TF-CNN, **(C)** MF-CNN.

The confusion matrix of the three deep learning network models were obtained. As shown in Figures 6, 7, the column represented the true label, and the row represented the predicted label. It can be seen that the probability of correct recognition of each motor imagery task by MF-CNN is higher than that of EEG-CNN and TF-CNN, and all the true positive values are greater than the true negative and false negative values for the three deep learning network models.

Finally, we calculated the kappa coefficient for each subject, and the mean results were shown in Table 1. The binary-class classification experiments obtained higher kappa values than the three-class classification experiment for all three deep learning models, and MF-CNN outperformed EEG-CNN and TF-CNN in the two experiments.

**TABLE 1:** Kappa values of the three deep learning models.

	EEG-CNN	TF-CNN	MF-CNN
Three-class classification	0.2662 ± 0.01	0.2536 ± 0.03	0.3559 ± 0.04
Binary-class classification	0.415 ± 0.04	0.3696 ± 0.02	0.5704 ± 0.02

## 4. Discussion

In this study, we performed feature fusion at the feature level to recognize the single upper limb motor imagery tasks by using deep learning approach. The dataset we used include three different types of movements, including forward extension of the arm, grasping the cup, and rotation of the wrist to the left. These are complex movements of the upper limb of the body and are commonly used in daily life. The accurate classification on the motor imagery of these three movements is of great significance in the application of BCI-based upper limb motor rehabilitation training. In this paper, the MF-CNN model was proposed to extract fusion features from the original EEG signal and corresponding time-frequency map. In the comparative experiment conducted on the single upper limb motor imagery dataset, MF-CNN model achieved better classification performance than two single CNN branches, EEGNET, ALEXNET, and CSP.

The EEG signal is non-stationary and non-linear (Yang et al., 2022). One of the most valuable methods for analyzing EEG signals is to transform them from one-dimensional time-domain signal to two-dimensional time-frequency map, which can concurrently combine the frequency feature in the time-domain and frequency-domain. The STFT and WT are the typical approaches for time-frequency analysis (Tabar and Halici, 2017; Yang et al., 2022). The STFT is obtained by adding a window on the basis of the Fourier transform. It has the ability of time-frequency analysis by using a fixed window function to analyze the signal segment. However, there are some shortcomings in the determination of the window function. If the window function is too narrow, the frequency domain analysis will be inaccurate; if it is too wide, the signal features in the time domain will be imprecise, affecting the time resolution. The WT is based on the Fourier transform but replacing the infinitely long triangular function base with a finite length and decaying wavelet base, and introduces scale and translation factors so that the resolution of the window function can change with the frequency characteristics. Compared with STFT, WT has the ability to obtain the local characteristics of the signal in both the time domain and the frequency domain (Khorrami and Moavenian, 2010). CWT offers a greater time-frequency resolution and can express the 3–5 s MI-EEG signal more precisely. Therefore, the EEG signal is transformed into a two-dimensional time-frequency map using the CWT method in the current study.

Previous studies based on deep learning usually used multi-channel stacked time-frequency maps as input to recognize motor imagery EEG (Dai et al., 2019). We have also tried this method, but could not obtain higher accuracy, only about 50% accuracy was achieved when using the time-frequency maps of C3, CZ, and C4. The reason for this may be that the aim of this study is to discriminative the motor imagery EEG of unilateral upper limbs, rather than the recognition of bilateral upper limb motor imagery in most studies. The difference between different actions in the unilateral upper limb motor imagery EEG is more minor (Ofner et al., 2017; Cho et al., 2021), thus it is challenging to obtain discriminative features with fewer channels. In order to make full use of the hidden information in the unilateral limb motor imagery EEG, we selected the EEG signals of 20 channels covering the sensorimotor cortex of the brain for analysis. However, it is not suitable to directly stack the 20-channel time-frequency maps as the input of TF-CNN. To solve this problem, we proposed to convert the time-frequency map based on the virtual channel after CSP spatial filtering. CSP could extract the spatial distribution components of each class from the multi-channel EEG data (Ramoser et al., 2000), and the virtual channel signal generated after spatial filtering contained the discriminative information between classes. The results shown in Figure 5 validated the effectiveness of this approach.

There are many successful applications for EEG signal classification using feature fusion methods of multi-modal signals. For instance, the feature fusions of facial pictures or sound signals with EEG signals have been proven to improve the classification accuracy of emotion recognition (Wagner et al., 2011; Xing et al., 2019). In the current study, the two-dimensional time-frequency maps converted by raw EEG signals were used as a supplement to the time-domain EEG signal. Since the time-frequency maps were calculated from the original EEG signals, this did not increase the complexity of the data acquisition and was suitable for rehabilitation training scenarios. In the processing of time-frequency images, TF-CNN was carried out from the perspective of image processing, which is quite different from the time-domain EEG signals processing of EEG-CNN. The information extracted from the two CNN branches were complementary, MF-CNN fused these information to make them complement each other. The results shown in Figure 6 validated that the classification accuracy of single upper limb motor imagery EEG could be improved by such fusion strategy.

## 5. Conclusion

In this study, we proposed a deep learning framework named MF-CNN for classifying EEG signals associated with single upper limb motor imagery. There are two branches in MF-CNN, which can simultaneously extract features from the original EEG signal and the two-dimensional time-frequency map, and fully learn the time domain and time-frequency domain features of the EEG signal. The binary-class and three-class classification test results on the unilateral upper limb motor imagery dataset demonstrated that the proposed MF-CNN can improve the classification performance of unilateral upper limb motor imagery EEG effectively.

## Data availability statement

Publicly available datasets were analyzed in this study. This data can be found here: http://gigadb.org/dataset/100788.

## Ethics statement

This study was reviewed and approved by the Institutional Review Board at Korea University (1040548-KU-IRB-17-181-A-2). The patients/participants provided their written informed consent to participate in this study. Written informed consent was obtained from the individual(s) for the publication of any potentially identifiable images or data included in this article.

## Author contributions

RZ and YC conceptualized the study, performed the majority of the experiments and analyses, made the figures, and wrote the first draft of the manuscript. ZX, LZ, YH, and MC performed some experiments, updated the figures, performed the statistics, and edited the manuscript. All authors approved the submitted version.
